# Correlations between Skin Condition Parameters and Ceramide Profiles in the Stratum Corneum of Healthy Individuals

**DOI:** 10.3390/ijms25158291

**Published:** 2024-07-29

**Authors:** Fuminari Akiyama, Natsumi Takahashi, Yuto Ueda, Shizuno Tada, Nobuyuki Takeuchi, Yusuke Ohno, Akio Kihara

**Affiliations:** 1 Taisho Pharmaceutical Co., Ltd., 3-24-1 Takada, Toshima-ku, Tokyo 170-8633, Japan; 2 Laboratory of Biochemistry, Faculty of Pharmaceutical Sciences, Hokkaido University, Kita 12-jo, Nishi 6-chome, Kita-ku, Sapporo 060-0812, Japan

**Keywords:** ceramide, lipids, skin, skin barrier, sphingolipids

## Abstract

Ceramides are essential lipids for skin barrier function, and various classes and species exist in the human stratum corneum (SC). To date, the relationship between skin conditions and ceramide composition in healthy individuals has remained largely unclear. In the present study, we measured six skin condition parameters (capacitance, transepidermal water loss, scaliness, roughness, multilayer exfoliation, and corneocyte cell size) for the SC of the cheeks and upper arms of 26 healthy individuals and performed correlation analyses with their SC ceramide profiles, which we measured via liquid chromatography–tandem mass spectrometry. In the cheeks, high levels and/or ratios of two free ceramide classes containing an extra hydroxyl group in the long-chain moiety and a protein-bound ceramide class containing 6-hydroxysphingosine correlated with healthy skin conditions. In contrast, the ratios of two other free ceramide classes, both containing sphingosine, and a protein-bound ceramide class containing 4,14-sphingadiene correlated with unhealthy skin conditions, as did shortening of the carbon chain of the fatty acid portion of two ceramide classes containing non-hydroxy fatty acids. Thus, our findings help to elucidate the relationship between skin conditions and ceramide composition.

## 1. Introduction

The skin functions as a permeability barrier (hereafter, skin barrier) that prevents the invasion of pathogens, allergens, and chemicals from outside and the loss of water and electrolytes from the body. Impaired skin barrier function causes or increases the risk of skin disorders such as infectious diseases, atopic dermatitis, and ichthyosis [1,2,3]. The skin barrier function is attracting attention not only in the medical field but also in cosmetics, since skin dryness is associated with skin roughness [4].

The stratum corneum (SC), which constitutes the outermost layer of the epidermis, plays a central role in skin barrier formation. In SC, two lipid structures—the lipid lamellae, which are multilayered lipid structures existing outside corneocytes, and the corneocyte lipid envelope (CLE), which covers the surfaces of corneocytes—play especially important roles [5,6,7,8]. The major components of the lipid lamellae are free (non-protein-bound) ceramides, cholesterol, and free fatty acids (FAs), while the CLE is primarily composed of protein-bound ceramides, which bind covalently to corneocyte surface proteins [6,7,8,9]. 

Ceramides are composed of a long-chain base (LCB) and an FA linked by an amide bond. Five types of LCBs (sphingosine [S], dihydrosphingosine [DS], 6-hydroxysphingosine [H], phytosphingosine [P], and 4,14-sphingadiene [SD]) and five types of FAs (non-hydroxy FA [N], α-hydroxy FA [A], ω-hydroxy FA [O], esterified ω-hydroxy FA [EO], and protein-bound FA [PB-]) exist in human ceramides (Supplementary Figure S1) [6,10]. Accordingly, human ceramides are classified into 25 classes based on the different combinations of FAs and LCBs, and each class is represented by a combination of the abbreviations for the FA and LCB (for example, ceramides composed of a non-hydroxy FA and sphingosine are NS) (Supplementary Figure S1). Of these, those with PB-type FAs (PB-S, PB-DS, PB-H, PB-P, and PB-SD) are protein-bound ceramides (the components of CLE), while the other 20 classes are free ceramides and constitute the lipid lamellae. Each ceramide class contains various ceramide species composed of LCBs and FAs with different carbon chain lengths and/or numbers of double bonds (in the case of FAs), and there are over 1500 ceramide species in the human SC [11]. Ceramides with EO-type FAs (EOS, EODS, EOH, EOP, and EOSD) are called ω-*O*-acylceramides (hereafter referred to as acylceramides). In acylceramides, linoleic acid is mainly esterified at the ω-position of the ω-hydroxy FA moiety [12]. Acylceramides are important for the formation and maintenance of the lipid lamellae [13,14]. Mutations of the genes involved in the production of acylceramides or protein-bound ceramides cause congenital ichthyosis [1,15,16,17,18,19,20,21,22,23].

Reduced ceramide levels, changes in ceramide class composition (decreases in NP, NH, EOS, EOH, and EOP and an increase in AS), and shortening of ceramide chains have been observed in patients with atopic dermatitis via thin-layer chromatography (TLC) and liquid chromatography coupled with mass spectrometry (LC-MS) analyses [24,25,26,27,28]. Correlations have also been found between the quantities of specific ceramide classes in the SC and skin barrier function parameters. For example, quantities of NH and NP are negatively correlated with transepidermal water loss (TEWL) and positively correlated with skin capacitance, whereas the reverse is true for NS and AS [26]. In addition, in patients with atopic dermatitis and psoriasis, reductions in the ratios of two specific ceramide classes (e.g., NP/NS, NH/NS, NP/AS, and NH/AS) in the SC have been observed, and these ratios have been shown to be negatively correlated with TEWL and positively correlated with skin capacitance [29]. Some ratios between two ceramide classes, such as NP/NS, have also been reported to correlate with cheek skin condition parameters (TEWL, skin capacitance, visual scaling score, and lightness), even in healthy individuals [29]. However, as the correlation analyses conducted so far in healthy individuals have focused only on free ceramide classes, the existence and strength of any correlations between quantities of protein-bound ceramides and skin condition parameters remain unknown. In addition, the quantitative analyses of free ceramides that have been conducted have mostly been done using LC-MS, not using LC coupled with tandem mass spectrometry (LC-MS/MS). 

In LC-MS measurements, ceramide species can only be identified based on the sum of the carbon chain lengths of the LCBs and FAs that constitute them. Therefore, in LC-MS analysis, it is not always possible to clarify whether the carbon chain length of the LCB or FA moiety specifically is correlated with the skin condition parameters. In contrast, LC-MS/MS analyses can quantify ceramide species by distinguishing the chain lengths of the constituent LCBs and FAs. A recent analysis using LC-MS/MS that focused on limited ceramide classes revealed shortening of the FA chain length of NS with an LCB chain length of C18, but not of NS with an LCB chain length of C20 or C22, in patients with atopic dermatitis [30]. Therefore, it is important to quantify ceramide species by distinguishing the chain lengths of the constituent LCBs and FAs via LC-MS/MS to clarify the correlations between the skin condition parameters and ceramides in more detail. We previously established an LC-MS/MS system that can comprehensively measure ceramide species [11]. In the present study, we focused on ceramides with the most major LCB chain length of C18 using our LC-MS/MS system and examined the correlations between both free and protein-bound ceramides and various skin condition parameters for human SC (capacitance, TEWL, multilayer exfoliation, corneocyte cell size, roughness, and scaliness).

## 2. Results

### 2.1. Differences in Skin Condition Parameters between the Cheeks and Upper Arms

**Figure 1:**
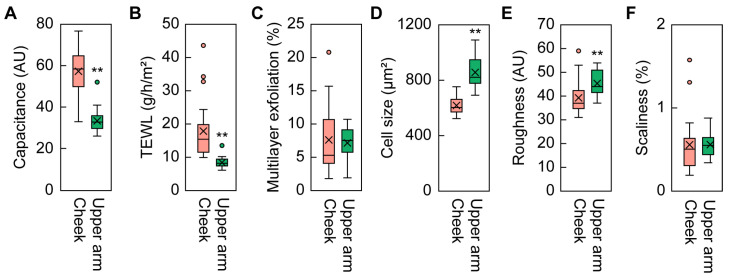
Skin condition parameters. The parameters ((**A**), capacitance; (**B**), transepidermal water loss [TEWL]; (**C**), multilayer exfoliation; (**D**), corneocyte cell size; (**E**), roughness; (**F**), scaliness) were measured for the cheeks and upper arms of healthy women (31–49 years old; n = 26) in winter. Boxes indicate the interquartile range (IQR), and lines in boxes represent the median. Whiskers indicate minimum and maximum values within 1.5 times the IQR. Circles and crosses represent outliers and means, respectively (** *p* < 0.01; Welch’s *t*-test). AU, arbitrary unit.

To reveal the relationship between the skin condition parameters and SC ceramide profile, we first measured the parameters (capacitance, TEWL, multilayer exfoliation, corneocyte cell size, roughness, and scaliness) for the cheeks and upper arms of 26 adult women (average age: 39.8 ± 5.3 years) with healthy skin during winter—the dry season in Japan. Capacitance represents the water content in the SC, with lower values indicating lower water content and reflecting dry skin. We found that the average capacitance value for the cheeks was about double that in the upper arms (Figure 1A). TEWL represents the amount of water that evaporates from the skin, and lower TEWL values indicate a stronger skin barrier. We found higher values of TEWL in the cheeks than in the upper arms (Figure 1B), which is consistent with previous studies [26,31]. The degree of multilayer exfoliation can be used to assess the condition of the SC: higher multilayer exfoliation values indicate rougher skin. These values were comparable between the cheeks and upper arms (Figure 1C). Corneocyte cell size is known to decrease with increased epidermal turnover rate, which can be caused by inflammation and skin roughness [32,33]. We observed that corneocyte cell sizes were larger in the upper arms than in the cheeks (Figure 1D), in agreement with previous studies [34]. Both skin roughness (reflecting the skin surface texture) and scaliness (reflecting the extent of shedding of small flakes of skin from the skin surface) are indicators of skin health, with lower values suggesting better skin condition. The average roughness value was slightly higher in the upper arms than in the cheeks, whereas scaliness was similar in both (Figure 1E,F). Overall, most of the parameters (except for cell size) showed greater variability between individuals for the cheeks than the upper arms.

### 2.2. Differences in Skin Condition Parameters between the Dry and Non-Dry Groups

**Figure 2:**
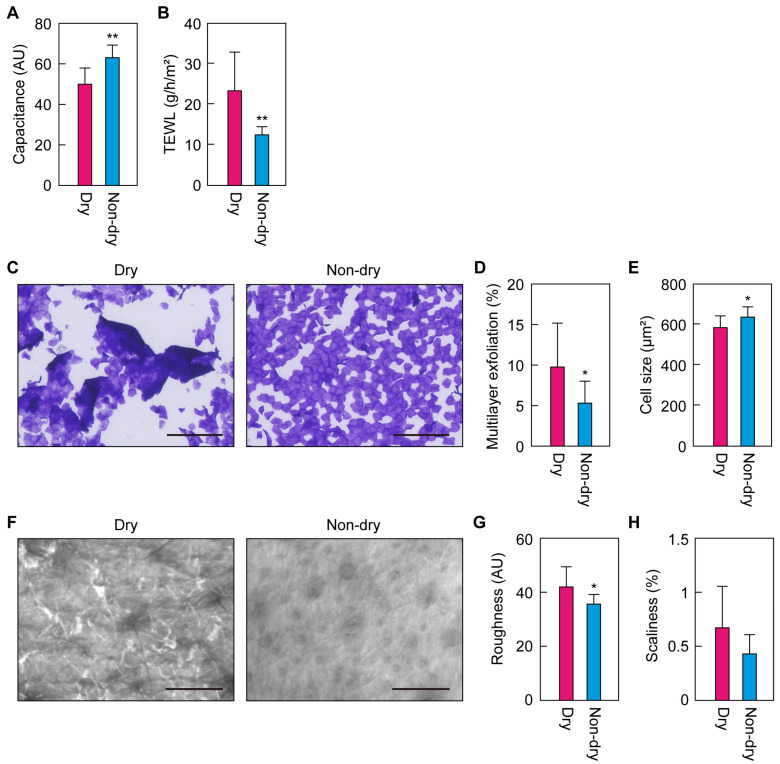
Skin condition parameters for the cheek in the dry and non-dry groups. The parameters ((**A**), capacitance; (**B**), transepidermal water loss [TEWL]; (**D**), multilayer exfoliation; (**E**), corneocyte cell size; (**G**), roughness; (**H**), scaliness) were measured in winter and were compared between the dry and non-dry groups (n = 13 each). Bars and whiskers indicate means and standard deviations (* *p* < 0.05; ** *p* < 0.01; Welch’s *t*-test). AU, arbitrary unit. (**C**) After collecting the stratum corneum by tape stripping, samples were subjected to brilliant green/gentian violet staining. The images are typical examples from each of the groups. Scale bars, 200 μm. (**F**) Images of the skin surface were obtained using Visioscan VC98. The images are typical examples from each of the groups. Scale bars, 1 mm.

To elucidate the effects of skin dryness on the skin condition parameters, we divided the 26 participants into two groups (13 each): non-dry, with higher cheek capacitance values, and dry, with lower cheek capacitance values. We then compared the other skin condition parameters for the cheeks between the two groups. The non-dry group had higher capacitance and lower TEWL values than the dry group (Figure 2A,B). Brilliant green/gentian violet (BG) staining revealed that, in the non-dry group, the SC was more likely to be detached as a single layer, with the shape and size of the corneocytes being more uniform and larger, respectively, than in the dry group, resulting in lower multilayer exfoliation values and higher corneocyte cell sizes (Figure 2C–E). The skin surface in the non-dry group was smoother than in the dry group and showed a lower tendency toward scaliness (Figure 2F–H). These results indicate that the non-dry group had more healthy skin than the dry group.

We also compared the parameters between the two groups for the upper arms but found no significant differences (Supplementary Figure S2). This is consistent with the above results showing that there was little individual variation in the parameters for the upper arms (Figure 1).

### 2.3. Ceramide Composition in the SC of the Cheeks and Upper Arms

**Figure 3:**
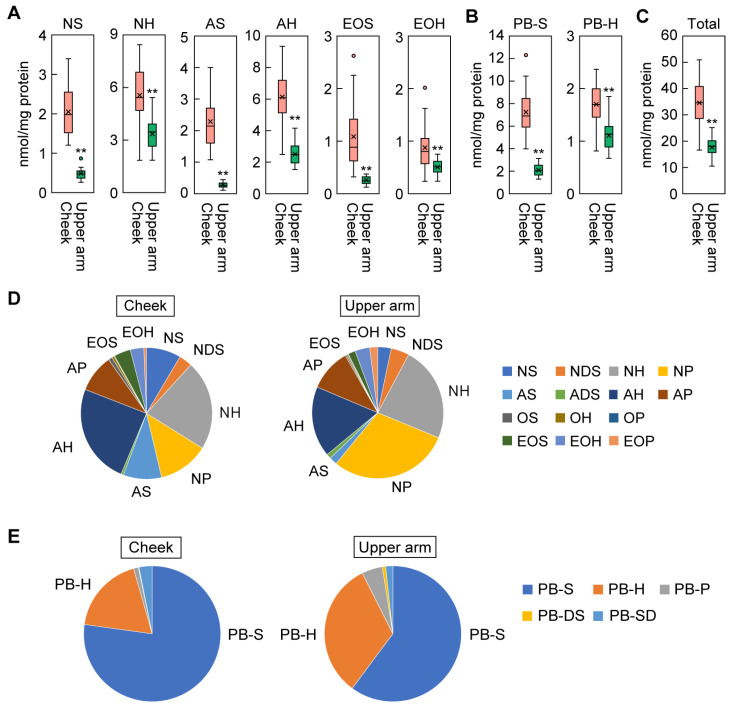
Ceramide profiles. Stratum corneum samples were collected from the cheeks and upper arms of healthy women (31–49 years old; n = 26) by tape stripping in winter, and ceramides were quantified via liquid chromatography coupled with tandem mass spectrometry. Quantities of free ceramides (**A**), protein-bound ceramides (**B**), and total ceramides (**C**) are shown in box-and-whisker plots. Boxes indicate the interquartile range (IQR), and lines in boxes represent the median. Whiskers indicate minimum and maximum values within 1.5 times the IQR. Circles and crosses represent outliers and means, respectively (** *p* < 0.01; Welch’s *t*-test). AU, arbitrary unit. The proportions of free ceramides (**D**) and protein-bound ceramides (**E**) comprised by each ceramide class are shown in pie charts.

To compare the ceramide profile of the SC of the cheeks and upper arms, SC samples were collected from both regions by tape stripping, after which the samples were subjected to lipid extraction and ceramide measurement via LC-MS/MS. Regarding free ceramides, there were substantial differences between the cheeks and upper arms in the levels of the ceramide classes NS, AS, and EOS, with average values being 4.2-, 8.0-, and 4.4-fold higher in the cheeks, respectively, than in the upper arms (Figure 3A and Supplementary Table S1). The next-largest differences were in the levels of NH, AH, and EOH (1.6-, 2.4-, and 1.7-fold higher in the cheeks, respectively, than in the upper arms). In contrast, the quantities of the DS- and P-type ceramide classes were comparable between the cheeks and upper arms (Supplementary Table S1). For the protein-bound ceramides, the quantities of PB-S and PB-H were 3.5- and 1.5-fold higher in the cheeks than the upper arms (Figure 3B). The total quantity of SC ceramides was about twice as high in the cheeks as in the upper arms (Figure 3C). Again, the inter-individual variation in these values was greater in the cheeks than in the upper arms (Figure 3A–C).

The ceramide class composition differed between the two regions. Of the free ceramide classes, AH was the most abundant in the cheeks, followed by NH, NP, AP, AS, NS, and EOS (Figure 3D). In contrast, in the upper arms, the order was NP, NH, AH, AP, NDS, EOH, NS, and AS. Thus, the proportion of NP was higher in the upper arms than in the cheeks, while the proportions of NS, AS, AH, and EOS were lower. These results are consistent with previous reports [35]. Protein-bound ceramide composition was also different between the two regions. PB-S was the most abundant protein-bound ceramide class in both the cheeks and the upper arms (Figure 3E), but the percentage was higher in the cheeks (77%) than in the upper arms (60%). In contrast, PB-H constituted a higher proportion in the upper arms (32.3%) than in the cheeks (18.4%). These results indicate that these two regions have characteristic ceramide compositions.

### 2.4. Differences in Ceramide Class Composition between the Dry and Non-Dry Groups

**Figure 4:**
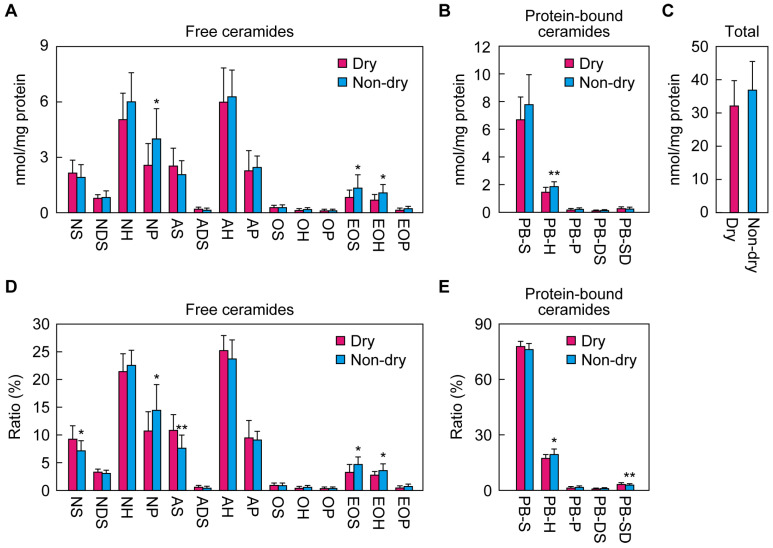
Ceramide class composition of cheek samples from the dry and non-dry groups. Quantities of ceramides ((**A**), free ceramide classes; (**B**), protein-bound ceramide classes; (**C**), total ceramides) and ratios of ceramide classes to total ceramides ((**D**), free ceramide classes; (**E**), protein-bound ceramide classes) in the cheeks in winter were compared between the dry and non-dry groups (n = 13 each). Bars and whiskers represent means and standard deviations (* *p* < 0.05; ** *p* < 0.01; Welch’s *t*-test).

We next compared the ceramide class composition in the cheeks between the dry and non-dry groups. Of the free ceramide classes, NP, EOS, and EOH were present in higher quantities in the non-dry group than the dry group, but no statistically significant differences between the two groups were observed for the other ceramide classes (Figure 4A). Regarding the protein-bound ceramides, the quantity of PB-H was greater in the non-dry group than the dry group (Figure 4B). The total quantity of ceramides was comparable between the two groups (Figure 4C).

The above absolute values for the SC ceramides were calculated by dividing the ceramide quantities by the protein quantities. Although we could quantify the ceramides via LC-MS/MS with little measurement error by using an internal standard, this was not the case for the protein quantification, mainly due to low levels of proteins in the tape stripping samples. As a result, the relatively large measurement error in the protein quantitation values led to large variation in the absolute values for the SC ceramides. To circumvent this, we next calculated the ratio of each ceramide class to the total quantity of ceramides. Using these values, we observed statistically significant differences between the dry and non-dry groups in NS, AS, and PB-SD, in addition to the significant differences in NP, EOS, EOH, and PB-H already seen in the absolute values (Figure 4D,E). The proportions of NS, AS, and PB-SD among total ceramides were higher in the dry group than in the non-dry group. In summary, there were differences in the quantities and/or ratios of specific ceramide classes to total ceramides in the cheeks between the dry and non-dry groups.

We then compared the ceramide class composition and total ceramide quantities in the upper arms between these groups, but we found no significant differences (Supplementary Figure S3). This is consistent with the above findings that there was little individual variation in skin condition parameters and ceramide levels for the upper arms (Figure 1 and Figure 3).

### 2.5. Correlation between Skin Condition Parameters and Ceramide Class Ratios

**Figure 5:**
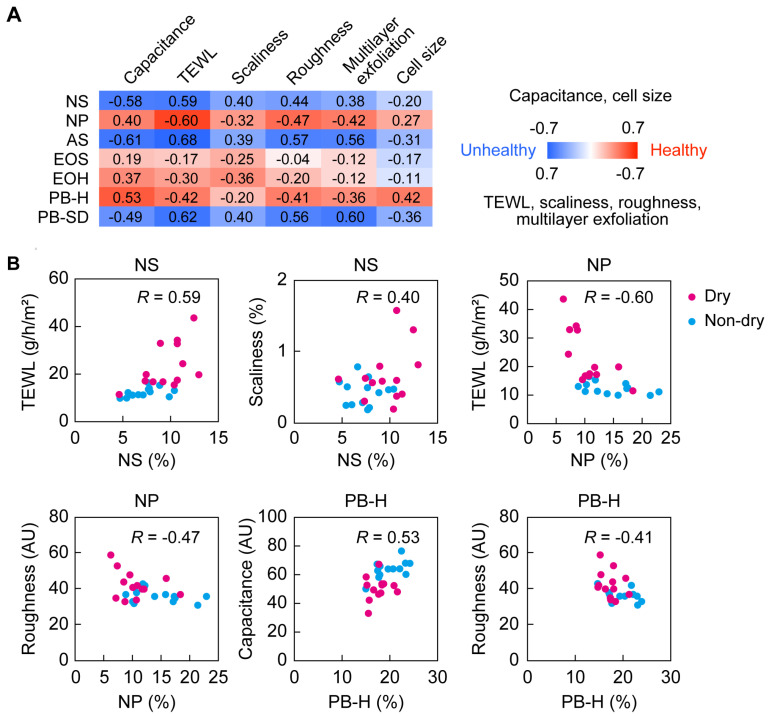
Correlations between ceramide class ratios and skin condition parameters. Correlations were analyzed between seven ceramide classes (NS, NP, AS, EOS, EOH, PB-H, and PB-SD) and six skin condition parameters (capacitance, transepidermal water loss [TEWL], scaliness, roughness, multilayer exfoliation, and corneocyte cell size) for samples taken from the cheeks in winter. (**A**) The strength of each correlation is indicated in the heatmap. Red and blue indicate correlations with healthy and unhealthy skin conditions, respectively. The numbers are the correlation coefficients (*R*). (**B**) Representative scatter plots of the ceramide class ratios and skin condition parameters (magenta, dry group; light blue, non-dry group).

To clarify the relationship between the skin condition parameters and ceramide profiles, we conducted analyses of the correlation between each skin condition parameter and the ceramide classes that differed between the dry and non-dry groups (NS, NP, AS, EOS, EOH, PB-H, and PB-SD; Figure 4). In this study, an absolute value of the correlation coefficient (|*R*|) of ≥0.3 was defined as a meaningful correlation. The capacitance was negatively correlated with NS, AS, and PB-SD and positively correlated with NP, EOH, and PB-H (Figure 5A,B). TEWL was positively correlated with NS, AS, and PB-SD and negatively correlated with NP, EOH, and PB-H. For the parameters related to the appearance of the skin (scaliness and roughness), scaliness was positively correlated with NS, AS, and PB-SD and negatively correlated with NP and EOH, while skin roughness was positively correlated with NS, AS, and PB-SD and negatively correlated with NP and PB-H. Regarding the parameters related to the state of the SC (multilayer exfoliation and corneocyte cell size), multilayer exfoliation was positively correlated with NS, AS, and PB-SD and negatively correlated with NP and PB-H, while corneocyte cell size was negatively correlated with AS and PB-SD and positively correlated with PB-H. In summary, NP, EOH, and PB-H were correlated with healthy skin conditions, while NS, AS, and PB-SD were correlated with unhealthy skin conditions. Although EOS showed no correlation at |*R*| ≥ 0.3 with any of the skin condition parameters, it did show weaker correlations with healthy skin conditions.

### 2.6. Correlations between Skin Condition Parameters and Chain Length of the FA Moiety of Ceramides

**Figure 6:**
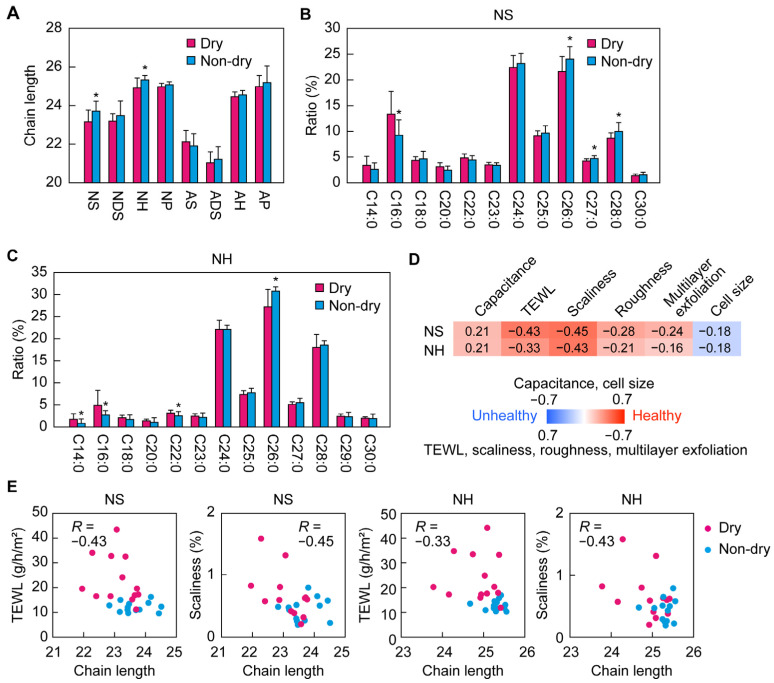
Ceramide chain lengths for the cheek in the dry and non-dry groups. Weighted averages of the chain lengths of free ceramide classes (**A**) and fatty acid composition of NS (**B**) and NH (**C**) for the cheeks in winter were compared between the dry and non-dry groups (n = 13 each). Bars and whiskers represent means and standard deviations (* *p* < 0.05; Welch’s *t*-test). (**D**) Analysis of correlations in the NS and NH classes between FA chain length and skin condition parameters (capacitance, transepidermal water loss [TEWL], scaliness, roughness, multilayer exfoliation, and corneocyte cell size) for the cheeks in winter. The strength of each correlation is indicated in the heatmap. Red and blue indicate correlation with healthy and unhealthy skin conditions, respectively. The numbers are the correlation coefficients (*R*). (**E**) Representative scatter plots of the fatty acid chain lengths of NS and NH and skin condition parameters (magenta, dry group; light blue, non-dry group).

In patients with atopic dermatitis, the carbon chain lengths (sum of the chain lengths of the LCBs and FAs) of ceramides are reported to be shorter than those in healthy individuals [26,27,28]. We next compared the chain lengths of the FA portions of ceramides between the dry and non-dry groups. We found that the chain lengths of NS and NH were shorter in the dry group than in non-dry group (Figure 6A). The weighted averages of the FA chain lengths of NS and NH in the non-dry group were 23.7 and 25.3, respectively, while in the dry group they were 23.2 and 24.9, respectively (Figure 6A). For NS, the ratio of C16:0 FA was higher in the dry group compared to the non-dry group, while those of C26:0, C27:0, and C28:0 FAs were lower (Figure 6B). In the case of NH, the ratios of C14:0, C16:0, and C22:0 FAs were higher in the dry group, while that of C26:0 FA was lower (Figure 6C).

Next, we examined the correlations between the weighted average FA chain lengths of NS and NH and the skin condition parameters. The FA chain lengths of NS and NH were negatively correlated with TEWL and scaliness (Figure 6D,E). These results suggest that longer chain lengths of the FA portions of NS and NH are correlated with healthy skin conditions.

### 2.7. Differences in Ceramide Class Composition between Summer and Winter

**Figure 7:**
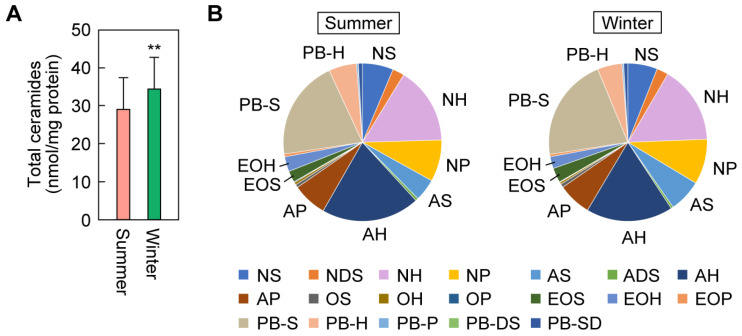
Differences in ceramide levels and class composition in the cheeks between seasons. Stratum corneum samples were collected from the cheeks of healthy women (31–49 years old; n = 26) by tape stripping in summer and winter, and ceramides were quantified via liquid chromatography coupled with tandem mass spectrometry. (**A**) Bars and whiskers represent means and standard deviations of total ceramide quantities (** *p* < 0.01; paired Student’s *t*-test). (**B**) The ratios of the ceramide classes are shown in pie charts.

To reveal the effects of the external environment on the quantities and composition of ceramides, we also measured cheek ceramides in summer (wet season) and compared them with those measured in winter (dry season; described above). We found that the total ceramide quantity in winter was 1.2-fold that in summer (Figure 7A). Among the ceramide classes, the ratios of AS and EOS were higher in the winter (1.5- and 1.3-fold, respectively) than in summer, while the ratios of AH and EOH were lower (0.9-fold and 0.8-fold, respectively) than in summer (Figure 7B). Thus, ceramide levels and composition differ between the seasons. The higher ceramide levels in winter imply an adaptation to the dry external environment.

## 3. Discussion

Several studies have reported changes in free ceramide profiles under skin pathologies such as atopic dermatitis and psoriasis [24,25,26,27,28,29,30]. However, knowledge about the relationship between ceramide profiles and skin condition parameters in healthy skin has been limited. In this study, we measured all ceramide classes (including protein-bound ceramides) via LC-MS/MS and examined the correlation between a large number of skin parameters (capacitance, TEWL, multilayer exfoliation, corneocyte cell size, roughness, and scaliness) and ceramide profile. We found that NP, EOS, EOH, and PB-H comprised a greater proportion of total ceramides in the non-dry group than the dry group (Figure 4), and higher ratios of NP, EOH, and PB-H were correlated with healthy skin, whereas those of NS, AS, and PB-SD were correlated with unhealthy skin (Figure 5). Changes in the quantities or ratios of free ceramides (NP, NS, AS, EOS, and EOH) have also been observed in patients with atopic dermatitis [24,25,26]. This indicates that such changes are generally observed not only in pathological conditions like atopic dermatitis but also in healthy individuals with unhealthy skin conditions, albeit to a lesser extent. However, since the relationship between protein-bound ceramides and skin condition has not previously been investigated, our finding that the ratios of PB-H and PB-SD constitute good indicators of skin condition is novel.

NP, EOH, and PB-H, which were especially strongly correlated with healthy skin conditions (Figure 5), commonly have one additional hydroxyl group in the LCB moiety compared to the ceramides that were correlated with unhealthy skin conditions (NS, AS, and PB-SD). We speculate that these hydroxyl groups strengthen the lipid–lipid interactions in the lipid lamellae via hydrogen bond formation, thereby enhancing the skin barrier function. Alternatively, the hydroxyl groups may interact with water, leading to water retention in the SC. Since acylceramides function in the formation and maintenance of the lipid lamellae [13,14], higher levels of EOH and EOS may stabilize the lipid lamellar structure. In contrast, the unhealthy ceramide PB-SD has a characteristic *cis*-double bond in the LCB moiety, and the bent structure of this *cis*-double bond may weaken lipid–lipid interactions.

Shortening of the chain length of NS has been reported in patients with atopic dermatitis [26,27,28]. Since in those studies only the total carbon chain length of both the LCB and the FA moieties combined was determined via LC-MS, it remained unclear whether it was the LCB or the FA moiety that was shortened. In this study, we revealed that shortening of the FA portion of ceramides is related to unhealthy skin conditions (Figure 6). The FA elongase ELOVL1 is responsible for the elongation of C22 and C24 acyl-CoAs to C24 and/or C26 acyl-CoAs [13,36]. In *ELOVL1*-mutated ichthyosis patients and in *Elovl1*-KO mice, shortening of the FA chain length of NS ceramides and reduction in acylceramide EOS levels were observed [13,37]. Based on these observations, we hypothesize that the expression levels of *ELOVL1* or the enzyme activity of the ELOVL1 protein were reduced in the epidermis of the dry group.

Most of the skin condition parameter values and the quantities of ceramide classes differed more between individuals (in terms of both the distribution and range of each value) in the cheeks than in the upper arms (Figure 1 and Figure 3). Compared to the upper arms, cheeks are more exposed to external stimuli such as dryness and UV radiation in general. It is possible that the degree of exposure to external stimuli varies substantially among individuals, leading to the larger individual differences in the cheeks.

In this study, we found that total ceramide levels were higher in the cheeks than in the upper arms (Figure 3C), contrary to a previous report [35]. This discrepancy could be due to differences in the measurement system (LC-MS vs. LC-MS/MS). The value reported previously represents the sum of all ceramide species, whereas that reported in this study represents only the sum of ceramides with a C18 LCB. Thus, it is possible that the levels of ceramides with LCBs with chain lengths other than C18 are lower in the cheeks than in the upper arms.

In this study, we revealed the relationship between the composition of ceramides containing a C18 LCB and skin conditions. In particular, we found for the first time that the protein-bound ceramides PB-H and PB-SD are good indicators for skin conditions. Our findings will be useful for the diagnosis of skin barrier abnormalities or skin health in the medical and cosmetic fields. In the future, analyses of ceramides containing LCBs other than C18 will be needed. In addition, elucidation of the molecular mechanism that causes the changes in ceramide composition induced under unhealthy skin conditions is important.

## 4. Materials and Methods

### 4.1. Subject

The skin condition parameter measurements and SC collection were performed on Japanese women (31–49 years old; n = 26) with healthy skin. These subjects were classified into two groups, a non-dry group (higher value) and a dry group (lower value), of 13 subjects each, based on the capacitance values of the samples from their cheeks.

### 4.2. Measurements of Skin Condition Parameters

After the subjects had rested for more than 20 min in a room with a temperature of 21 ± 1 °C and a humidity of 50 ± 5%, the skin condition parameters were measured on the cheeks and inner upper arms, as described below. TEWL was measured using a Tewameter TM300 (Courage + Khazaka Electronic, Köln, Germany). Measurements were conducted once per second for 60 s at the same site, and the average of the 10 measurements with the lowest standard deviation of a total of 60 values were used. The capacitance was measured using a Corneometer CM825 (Courage + Khazaka Electronic) five times at the same site, and the average of the three measurements between the maximum and minimum values were used. Scaliness and roughness were analyzed with a Visioscan VC98 (Courage + Khazaka Electronic). After images of the skin surface were obtained, each value was calculated using SELS software (version 2.6.4.0) for an area of skin of approximately 12 mm × 9.5 mm.

Multilayer exfoliation and corneocyte cell size were measured as follows. The SC samples were peeled off with cellophane tape (24 mm × 50 mm, Nichiban, Tokyo, Japan) and transferred to a slide glass and stained with BG as described previously [38]. Images were captured using an optical microscope (LV100ND, Nikon, Tokyo, Japan), and the degree of multilayer exfoliation and corneocyte cell size were analyzed using the Corneocytometry software (version 2, Niigata S-Labo, Niigata, Japan). The degree of multilayer exfoliation was calculated as the ratio of the area of the multilayered cells to total corneocyte area, and the average of three images was used. Corneocyte cell size was calculated as the average of the area of corneocytes obtained from three images (30 corneocytes per image).

### 4.3. Ceramide Measurement via LC-MS/MS

SC samples were collected from the other side of the body from the first collection sites for the measurements of multilayer exfoliation and corneocyte cell size using film masking tape No. 465 (25 mm × 50 mm; Teraoka Seisakusho, Tokyo, Japan) as described previously [39]. Lipids were prepared from the tape strip, and ceramides were measured via LC-MS/MS essentially as described previously [39]. Briefly, the film masking tape (5 mm × 10 mm) was suspended in 400 µL of methanol, and the following deuterium (*d*)-labeled ceramides all purchased from Avanti Polar Lipids (Alabaster, AL, USA) were added as internal standards: 0.5 pmol *N*-palmitoyl(*d*_9_) D-*erythro*-sphingosine (*d*_9_-C16:0 NS), 1 pmol *N*-palmitoyl(*d*_9_) dihydrosphingosine (*d*_9_-C16:0 NDS), 5 pmol *N*-palmitoyl (*d*_9_) D-*ribo*-phytosphingosine (*d*_9_-C16:0 NP), 5 pmol *N*-palmitoyl(*d*_9_) 6-(*R*)-hydroxysphingosine (*d*_9_-C16:0 NH), 0.5 pmol *N*-(2′-(*R*)-hydroxypalmitoyl(*d*_9_)) D-*erythro*-sphingosine (*d*_9_-C16:0 AS), 0.5 pmol *N*-(2′-(*R*)-hydroxypalmitoyl(*d*_9_)) D-*erythro*-dihydrosphingosine (*d*_9_-C16:0 ADS), 2 pmol *N*-(2′-(*R*)-hydroxypalmitoyl(*d*_9_)) D-*ribo*-phytosphingosine, (*d*_9_-C16:0 AP), 2 pmol *N*-(2′-(*R*)-hydroxypalmitoyl(*d*_9_)) 6-(*R*)-hydroxysphingosine (*d*_9_-C16:0 AH). After sonication at room temperature for 5 min, the tape was removed and centrifugated (2600× *g*, room temperature, 3 min). The supernatant was transferred to another glass tube and dried (for free ceramide measurements). The remaining SC pellet was suspended in 1 mL of methanol, divided into two moieties (one for protein-bound ceramide measurement and the other for protein quantification). Protein-bound ceramides were extracted as follows. First, 400 µL of methanol was added to the samples and mixed. After centrifugation (2600× *g*, room temperature, 3 min), the supernatant was removed. The same procedure was repeated twice. Then, 400 µL of 95% methanol was added to the pellet, followed by incubation (60 °C, 2 h), centrifugation (2600× *g*, room temperature, 3 min), and removal of the supernatant. After repeating this procedure, the pellet was suspended in 400 µL of 1 M potassium hydroxide/95% methanol containing internal standards (*d*_9_-C16:0 AS, 1 pmol; *d*_9_-C16:0 ADS, 0.25 pmol; *d*_9_-C16:0 AH, 1 pmol; *d*_9_-C16:0 AP, 0.25 pmol) and incubated at 60 °C for 2 h. The samples were neutralized with 400 µL of 1 M acetic acid, and 400 µL of chloroform was added to the samples and vigorously mixed (room temperature, 1 min). After centrifugation (2600× *g*, room temperature, 3 min), the lower layer containing protein-bound ceramides was collected and dried. Free ceramide and protein-bound ceramide samples were dissolved in 100 µL and 50 µL of chloroform/methanol (1:2, *v*/*v*), respectively, and a 5 µL aliquot was used for the LC-MS/MS analyses. LC-MS/MS was performed using an LC-coupled triple quadrupole mass spectrometer (Xevo TQ-S; Waters, Milford, MA, USA) equipped with a reverse-phase column (ACQUITY UPLC CSH C18 column; particle size, 1.7 µm; inner diameter, 2.1 mm; length, 100 mm; Waters). The conditions of LC separation, electrospray ionization, and multiple reaction monitoring mode measurement using MS/MS as well as the quantification method were as described previously [39]. Of the ceramides containing C18 LCB, we selected 115 free ceramide and 36 protein-bound ceramide species, each covering more than 95% of the total quantity of each of the categories, as described previously [40].

Protein quantification was performed as follows. After free ceramide extraction, the SC pellets were dried and suspended in 45 µL of 0.1 M NaOH + 1% SDS solution. After incubation at 60 °C for 2 h, 2.5 µL of 2 M HCl was added for neutralization, followed by the addition of 2.5 µL of 1 M Tris-HCl (pH 6.8). The protein solution thus obtained was diluted 10-fold with water, and protein quantification was performed using the Micro BCA protein assay kit (Thermo Fisher Scientific, Waltham, MA, USA) according to the manufacturer’s instructions.

### 4.4. Statistical Analysis

The creation of bar graphs and box-and-whisker plots and the statistical analysis were performed using Microsoft Excel (version 2202, Microsoft, Redmond, WA, USA). Student’s *t*-test was used to examine the significance of differences in the ceramide levels between seasons, and Welch’s *t*-test was used to examine the significances of differences in other data. *p*-values of <0.05 were considered significant.

## Data Availability

All data are included in the article and Supplementary Materials.

## Supplementary Materials

The supporting information can be downloaded at: https://www.mdpi.com/article/10.3390/ijms25158291/s1.
