# Supplementary material for: Correlations between Skin Condition Parameters and Ceramide Profiles in the Stratum Corneum of Healthy Individuals
Source: Int J Mol Sci. 2024 Jul 29;25(15):8291. doi: 10.3390/ijms25158291 (PMC11311646; doi:10.3390/ijms25158291)
Supplement: Supplementary file 1 [file ijms-25-08291-s001.zip › Supplementary Figures.pdf]

A

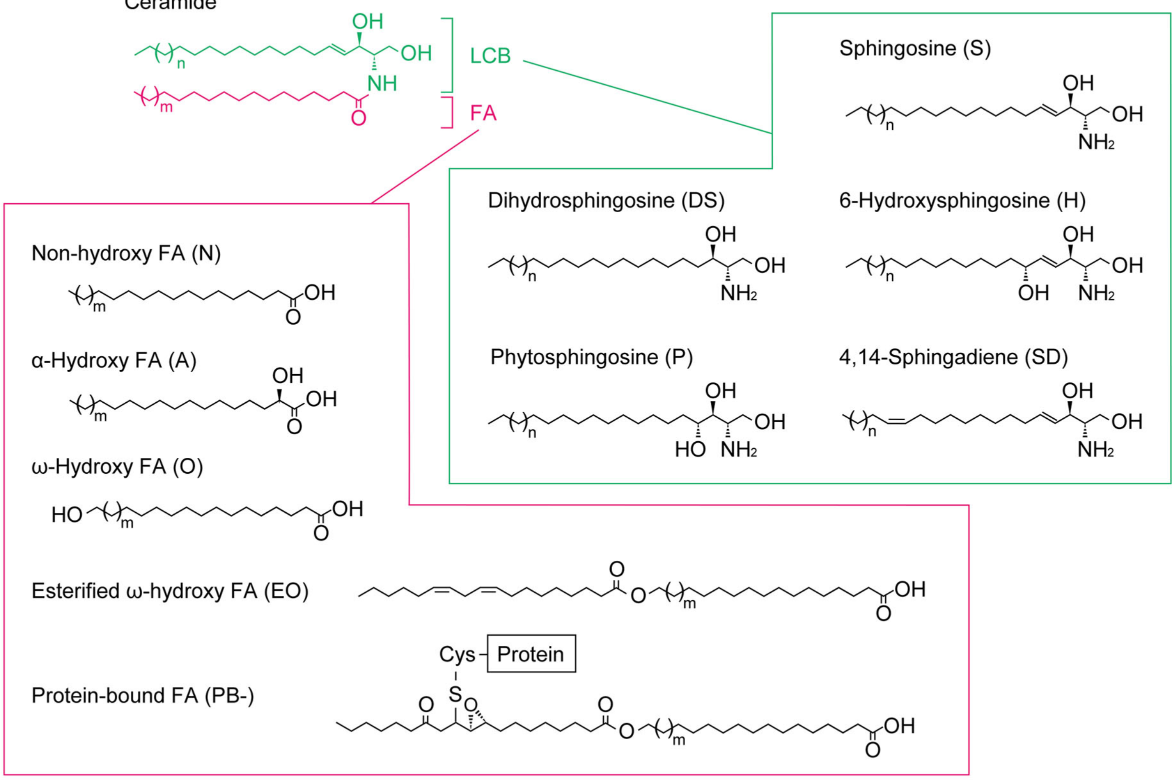

B

LCB \ FA	Non-hydroxy (N)	α-Hydroxy (A)	ω-Hydroxy (O)	Esterified ω-hydroxy (EO)	Protein-bound (PB-)
Dihydrosphingosine (DS)	NDS	ADS	ODS	EODS	PB-DS
Sphingosine (S)	NS	AS	OS	EOS	PB-S
Phytosphingosine (P)	NP	AP	OP	EOP	PB-P
6-Hydroxysphingosine (H)	NH	AH	OH	EOH	PB-H
4,14-Sphingadiene (SD)	NSD	ASD	OSD	EOSD	PB-SD

Non-acylated ceramides (N, A, O) | Acylceramides (EO) | Protein-bound ceramides (PB-)

Free ceramides (N, A, O, EO)

Supplementary Figure S1. Structures and nomenclature of ceramide classes. The structures of the LCBs and FAs constituting human ceramides (**A**) and the abbreviations used for the ceramide classes (**B**) are shown.

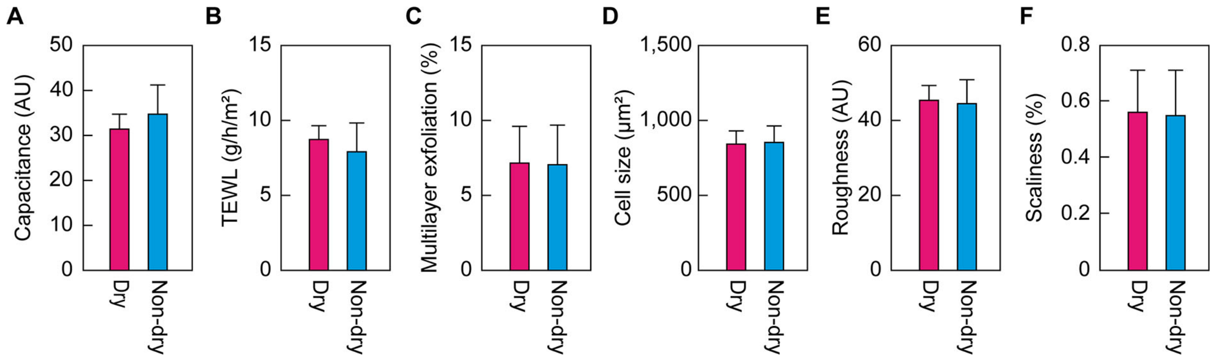

Supplementary Figure S2. Skin condition parameters in the upper arms in the dry and non-dry groups. Skin condition parameters (**A**, capacitance; **B**, TEWL; **C**, multilayer exfoliation; **D**, corneocyte cell size; **E**, roughness; **F**, scaliness) for the upper arms in winter were measured and compared between the dry and non-dry groups (n = 13 each). Bars and whiskers represent means and standard deviations. AU, arbitrary unit.

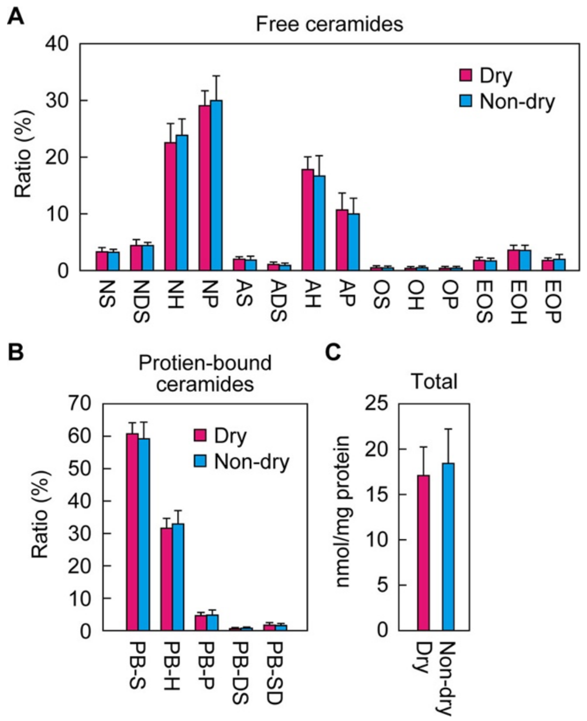

Supplementary Figure S3. Ceramide class composition for the upper arms in the dry and non-dry groups. The ratios of ceramide classes to total ceramides (**A**, free ceramide classes; **B**, protein-bound ceramide classes) and total quantities of ceramides (**C**) in the upper arms in winter were compared between the dry and non-dry groups (n = 13 each). Bars and whiskers represent means and standard deviations.
