# Supplementary material for: Correlations between Skin Condition Parameters and Ceramide Profiles in the Stratum Corneum of Healthy Individuals
Source: Int J Mol Sci. 2024 Jul 29;25(15):8291. doi: 10.3390/ijms25158291 (PMC11311646; doi:10.3390/ijms25158291)
Supplement: Supplementary file 1 [file ijms-25-08291-s001.zip › Supplementary Table.pdf]

Supplementary Table S1. Quantities of ceramide classes in winter

	Cheeks	Upper arms
NS	2.04 ± 0.63	0.47 ± 0.13
NDS	0.82 ± 0.24	0.65 ± 0.19
NH	5.53 ± 1.54	3.36 ± 0.96
NP	3.26 ± 1.58	4.20 ± 1.01
AS	2.29 ± 0.87	0.28 ± 0.09
ADS	0.16 ± 0.06	0.17 ± 0.07
AH	6.12 ± 1.62	2.49 ± 0.71
AP	2.36 ± 0.85	1.49 ± 0.51
OS	0.26 ± 0.14	0.05 ± 0.02
OH	0.15 ± 0.08	0.04 ± 0.01
OP	0.05 ± 0.03	0.03 ± 0.01
EOS	1.09 ± 0.60	0.25 ± 0.07
EOH	0.87 ± 0.42	0.51 ± 0.13
EOP	0.18 ± 0.11	0.27 ± 0.08
PB-S	7.25 ± 1.93	2.06 ± 0.45
PB-H	1.71 ± 0.39	1.10 ± 0.26
PB-P	0.11 ± 0.05	0.17 ± 0.05
PB-DS	0.01 ± 0.01	0.03 ± 0.01
PB-SD	0.30 ± 0.10	0.06 ± 0.01
Total	34.5 ± 8.26	17.67 ± 5.25
